# Sequencing and Characterisation of Complete Mitochondrial DNA Genome for *Trigonopoma pauciperforatum* (Cypriniformes: Cyprinidae: Danioninae) with Phylogenetic Consideration

**DOI:** 10.21315/tlsr2020.31.1.7

**Published:** 2020-04-07

**Authors:** Hung Hui Chung, Leonard Whye Kit Lim, Yunshi Liao, Tommy Tsan-Yuk Lam, Yee Ling Chong

**Affiliations:** 1 Faculty of Resource Science and Technology, Universiti Malaysia Sarawak, 94300 Kota Samarahan, Sarawak, Malaysia; 2 School of Public Health, The University of Hong Kong, Sassoon Road, Pokfulam, Hong Kong

**Keywords:** *Trigonopoma pauciperforatum*, Mitogenome, Gene arrangement, Light strand origin, Phylogenetic analysis, *Trigonopoma pauciperforatum*, Mitogenom, Urutan gen, Asal rantaian ringan, Analisa filogenetik

## Abstract

The *Trigonopoma pauciperforatum* or the redstripe rasbora is a cyprinid commonly found in marshes and swampy areas with slight acidic tannin-stained water in the tropics. In this study, the complete mitogenome sequence of *T. pauciperforatum* was first amplified in two parts using two pairs of overlapping primers and then sequenced. The size of the mitogenome is 16,707 bp, encompassing 22 transfer RNA genes, 13 protein-coding genes, two ribosomal RNA genes and a putative control region. Identical gene organisation was detected between this species and other family members. The heavy strand accommodates 28 genes while the light strand houses the remaining nine genes. Most protein-coding genes utilise ATG as start codon except for COI gene which uses GTG instead. The terminal associated sequence (TAS), central conserved sequence block (CSB-F, CSB-D and CSB-E) as well as variable sequence block (CSB-1, CSB-2 and CSB-3) are conserved in the control region. The maximum likelihood phylogenetic tree revealed the divergence of *T. pauciperforatum* from the basal region of the major clade, where its evolutionary relationships with *Boraras maculatus*, *Rasbora cephalotaenia* and *R. daniconius* are poorly resolved as suggested by the low bootstrap values. This work contributes towards the genetic resource enrichment for peat swamp conservation and comprehensive in-depth comparisons across other phylogenetic researches done on the *Rasbora*-related genus.

## Abstract (ms)

*T. pauciperforatum* atau *rasbora redstripe* adalah cyprinid yang biasa ditemui di kawasan rawa dan berpaya dengan air bersifat tannin-asid di tropik. Dalam kajian ini, urutan mitogenom lengkap *T. pauciperforatum* pertama kali diasingkan dalam dua bahagian dengan menggunakan dua pasang primer yang bertindih dan kemudian bahagianbahagian ini masing-masing dijujukan. Saiz mitogenom yang dikenal pasti adalah 16,707 bp, merangkumi 22 gen tRNA, 13 gen protein, dua gen RNA ribosom dan *control region*. Komposisi gen yang dikesan adalah sejajar antara spesies ini dan ahli genus rasbora yang lain. Rantaian berat menampung 28 gen manakala rantaian ringan menempatkan sembilan gen yang lain. Kebanyakan gen protein menggunakan ATG sebagai *codon* permulaan kecuali untuk gen COI yang menggunakan GTG. Urutan bersekutu terminal (TAS), blok urutan pusat (CSB-F, CSB-D dan CSB-E) serta blok turutan berubah (CSB-1, CSB-2 dan CSB-3) turut terpelihara dalam mitogenom ini. Pokok filogenetik mendedahkan perbezaan dari *T. pauciperforatum* dari bahagian *basal clade*, di mana hubungan evolusi dengan *Boraras maculatus*, *Rasbora cephalotaenia* dan *R. daniconius* tidak dapat dikenal pasti dengan baik seperti yang dicadangkan oleh nilai *bootstrap* yang rendah. Kerja ini menyumbang kepada pengayaan sumber genetik untuk pemuliharaan tanah gambut dan perbandingan yang komprehensif merentasi penyelidikan filogenetik seterusnya dilakukan pada genus berkaitan dengan Rasbora.

**Highlights**

- *T. pauciperforatum* complete mitogenome sequence isolated using two primer pairs targeting overlapping regions.
- Identical gene organisation between this species and other Rasbora counterparts.
- *T. pauciperforatum* diverged from the basal clade, where its relationships with *B. maculatus* and *R. daniconius* remains poorly resolved.

## INTRODUCTION

The redstripe rasbora (*Trigonopoma pauciperforatum*) (Weber & de Beaufort 1916) is grouped under the subfamily Danioninae in the Cyprinidae family. It has the distinctive thick striking red neon stripe which aligns in parallel to its spine, starting from the side of its jaw, crossing upper part of the eye and up till before its tail fin (Weber & de Beaufort 1916). Its greyish brown streamline body are equipped with smoke-grey fins, white belly as well as a fork-shaped caudal fin tail (Ward 2003). Females have bigger bellies than males in general because this species is egg-spawning fish. This red-striped rasbora fish can be found abundantly in school around stagnant fresh waters (rivers, drainages, lakes and streams) of South East Asia, including Peninsular Malaysia, Sarawak and Sumatra (Ward 2003). The type locality of this species is Sumatra. Their natural habitat has heavily grown and overhanging vegetation with minimal lighting. The diet of this fish is mainly made up of zooplankton, larvae and insects. Adult fish can grow up to the length of >6 cm (Ward 2003).

The *T. pauciperforatum* is a popular ornamental aquarium fish often mistaken for the Glowlight Tetra (*Hemigrammus erythrozonus*) (Durbin 1909) due to their high morphological similarities but they are distinguishable by the much brighter red stripe and the absence of adipose fin in the Redline Rasbora (Durbin 1909; Weber & de Beaufort 1916; Ward 2003). Due to their extremely selective breeding behaviour, breeding them in aquarium conditions is not easy and the success rate is higher when they are placed in school of 6 to 10 (Ward 2003). Adult females scatter their eggs all over overgrown vegetation before the adult males are stimulated release sperms to fertilise the eggs during the action of tailing the females. Egg hatching occurs within 1 to 2 days post fertilisation and the fry can swim freely within 3 to 5 days (Ward 2003). The lifespan of this fish ranges from 3 to 5 years with good care and maintenance under the following conditions: pH 6.2 to 7.0, 0 to 6-degree hardness and 22.7°C to 26°C (Ward 2003).

The *T. pauciperforatum* was previously classified under the genus *Rasbora.* The *Rasbora* genus encompasses a large group of diversified freshwater fishes, making it the most species-enriched genus (87 species as of 2015) in the Cyprinidae family (Fricke *et al.* 2018). The classification of the *Rasbora* genus possesses complications as it is known as the catch-all group lacking synapomorphies or shared derived characters (Brittan 1954; Kottelat & Vidthayanon 1993; Liao *et al.* 2010; Tang *et al.* 2010). The eight *Rasbora* species complexes defined by Brittan (1954) had been revised recurrently over the years by various researchers (Kottelat & Vidthayanon 1993; Siebert & Guiry 1996; Kottelat 2005; Liao *et al.* 2010) with some new genera being introduced and till now majority of them still hold firm on the *Rasbora sensu lato* concept by Brittan (1954) which encompasses all the new genera created. Yet, most of the *Rasbora* species lack the distinctive characters to form a monophyletic clade of its own both morphologically (Liao *et al.* 2010) and molecularly (mitochondrial COI, Cytb and nuclear RAG1) (Kusuma *et al.* 2016).

The use of *Rasbora* species in genetic research is picking up its pace recently with the discovery of their potential as ecotoxicology models (Lim *et al.* 2018; Wijeyaratne & Pathiratne 2006). To date, only nine *Rasbora* species (namely *R. argyrotaenia*, *R. sumatrana*, *R. trilineata*, *R. aprotaenia*, *R. steineri*, *R. lateristriata*, *R. daniconius*, *R. borapetensis* and *R. cephalotaenia*) and four other species previously classified under the *Rasbora* genus (*Rasboroides vaterifloris*, *Trigonostigma heteromorpha*, *T. espei* and *Boraras maculatus*) (Miya 2009; Tang *et al.* 2010; Chang *et al.* 2013; Ho *et al.* 2014; Zhang *et al.* 2014; Kusuma & Kumazawa 2015; Kusuma *et al.* 2017) had their mitochondrial genomic sequences published out of the total 87 species discovered thus far (Fricke *et al.* 2018), a mere 14.94%. The genus *T. pauciperforatum* resides in (*Trigonopoma*) contains only two species thus far, where its sole genus counterpart is *T. gracile*. To the best of our knowledge, *T. pauciperforatum* is the only species from this genus that have had its mitogenome sequenced and this accounts for the urgency to unravel more about the mitogenomes of its genus as well as natural habitat counterparts in order to obtain a bigger picture of the genetic biodiversity in the peat swamp for conservation purposes (Chen *et al.* 2016; Sule *et al.* 2018). On the other hand, the phylogenetic data based on whole mitogenome sequences of this species provides opportunities for comprehensive comparison of the phylogenetic tree constructed based on morphologies (Liao *et al.* 2010).

Thus, this study had shed light on the landscape of the complete mitochondrial genome of *T. pauciperforatum* beside further dissecting on the genetic contents and revealing the molecular phylogenetic relationship across 13 other closely related members of the Danioninae subfamily (from *Rasbora* genus and other species previously classified under *Rasbora* genus). This study also contributes towards the genetic resource enrichment for peat swamp conservation (Sule *et al*. 2018) and comprehensive in-depth comparisons across other phylogenetic researches (Liao *et al.* 2010; Kusuma *et al.* 2016) done on the *Rasbora*-related genus.

## MATERIALS AND METHODS

### Sampling and Genomic DNA Extraction

The *T. pauciperforatum* specimen was collected from Matang River, Sarawak, Malaysia (1.5755° N, 110.2990° E) with the permit issued by Sarawak Forestry Department (permit number: NCCD.94047(Jld13)-178). Adult fish was sacrificed humanely using Tricane^TM^ as anaesthetics with permission from Universiti Malaysia Sarawak Animal Ethics Committee (reference number: UNIMAS/TNC(PI)-04.01/06-09(17)). The muscle tissues were harvested from the fish body before subjecting to storage in 95% ethanol. The genomic DNA was extracted using CTAB method (Thomas *et al.* 2010).

### Primers Design, Long-PCR Amplification and DNA Sequencing

A total of two pairs of primers were designed based on the multiple alignment outcomes from the complete mitochondrial genome of four closely related *Rasbora* species including *R. argyrotaenia*, *R. sumatrana*, *R. trilineata* and *R. aprotaenia*. The primer pairs (Table 1) were designed to amplify two large fragments from the mitochondrial genome with overlapping of at least 2 kb at both ends of fragments to ensure good sequencing reads. The complete mitochondrial genome of *T. pauciperforatum* was assembled by joining the two large amplicon fragments and trimming overlapping sequences.

Long-Polymerase Chain Reaction (Long-PCR) was conducted using Bio-Rad T-100 Thermal Cycler in 20 μL total reaction volume encompassing 0.4 μL 10 μM forward and reverse primer each, 1.6 μL 2.5mM dNTP, 2.0 μL 10X PCR buffer (with Mg^2+^), 2.5 U high-fidelity *Taq* polymerase, 14.6 μL nucleasefree water and 0.8 μL genomic DNA extract orchestrated under conditions: one cycle of pre-denaturation at 94°C for 2 min, followed by 35 cycles of denaturation, annealing and extension at 94°C (30 s), primer-specific temperature (30 s) and 72°C (5 min) respectively and a final extension cycle at 72°C for 5 min. Agarose gel electrophoresis was performed to size separate the amplicons on 1% agarose gel for visualisation under UV light. PCR purification was done prior to pair-ended short-read DNA sequencing on Illumina HiSeq 4000 System (BGI, Hong Kong). Sequencing reads are quality-checked, adaptor-trimmed using cutadapt (Martin 2011) and assembled into the complete genome sequences using *de novo* assembler SPAdes (Bankevich *et al.* 2012).

### Mitochondrial Genome Characterisation and Gene Analysis

The mitochondrial genome map was constructed using MitoFish (Iwasaki *et al.* 2013) (http://mitofish.aori.u-tokyo.ac.jp/annotation/input.html). Using MEGA 7.0 (Kumar *et al.* 2016), the protein-coding genes were subjected to translation into amino acid sequences to amend truncated or premature stop codons to ensure their functionalities. The codon usage was determined using MEGA 7.0 (Kumar *et al.* 2016) whereas the nucleotide composition was calculated using DNA nucleotide counter (Heracle BioSoft 2014). All anti-codons of tRNA genes were identified using default search mode of the tRNA-scan SE v. 2.0 software (Lowe & Chan 2016) (http://lowelab.ucsc.edu/cgi-bin/tRNAscan-SE2.cgi). The L-strand origin (O_L_) determined thru sequence homology was then subjected to secondary structure visualisation using RNA structure 6.0 (Reuter & Mathews 2010). All DNA sequences forming the complete mitochondrial genome was deposited into the GenBank database via the Sequin software (http://www.ncbi.nlm.nih.gov/Sequin/).

### Phylogenetic Tree Construction

The raw data for phylogenetic analysis was collected from GenBank database which includes 13 other closely related members of the Danioninae subfamily (from *Rasbora* genus and other species previously classified under *Rasbora* genus) with complete mitochondrial genomic DNA available publicly; *Acheilognathus typus* and *Danio rerio* were selected as the outgroup. A total of 12 protein-coding genes (except for ND6 due to its high heterogeneity (Miya & Nishida 2000) were concatenated to one single fasta format entry for each species to be analysed by first conducting multiple sequence alignment using CLUSTALW in MEGA 7.0. A model test was performed using MEGA 7.0 prior to phylogenetic tree construction and the best suited model determined, the GTR+G (General Time Reversible model with Gamma distributed rates among sites) was employed via Maximum Likelihood (ML) analysis with bootstrap of 1000 replicates. The resultant phylogenetic tree was viewed using FigTree v1.4.2.

## RESULTS AND DISCUSSION

### Mitochondrial DNA Genome Structure

**Figure 1:**
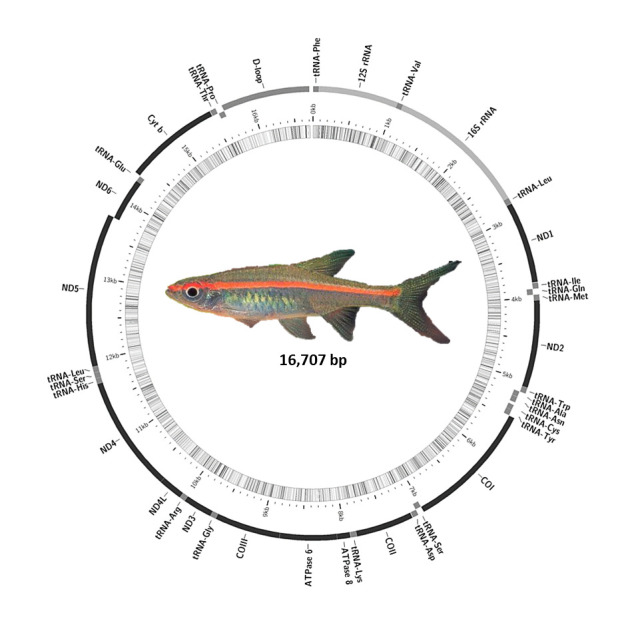
Circular genome map of *T. pauciperforatum*. Genes encoded on heavy and light strand are depicted in outer and inner circle respectively. The inner ring displays the GC percent per every 5 bp where the darker lines represent higher GC percent. The size of the complete mitogenome of *T. pauciperforatum* is 16,707 bp with the contribution from 22 tRNA genes, 13 protein-coding genes, two rRNA genes and a control region.

The size of the complete mitochondrial genome of *T. pauciperforatum* is 16,707 bp with the inclusion of 22 tRNA genes, 13 protein-coding genes, two rRNA genes and a control region (Fig. 1, Table 2). The complete mitochondrial genome sequence was deposited in the GenBank database with the assigned accession number MK034301. The heavy strand (H-strand) of the mitochondrion carries a total of 28 genes whereas the remaining are housed on the light strand (L-strand). All 4 overlaps detected from the entire mitochondrial genome are found on the H-strand. The greatest overlap (7 bp) was observed in both between genes ATP8 and ATP6 as well as between genes ND4L and ND4. The lengthiest intergenic spacer (34 bp) was detected between genes tRNA^Asn^ and tRNA^Cys^.

The overall A+T content of the mitochondrial genome (60.0%) is much greater than G+C content (40.0%) (Table 3) which is similar to *Cobitis lutheri*, *R. borapetensis* and *R. steineri* (Cui *et al.* 2013; Zhang *et al.* 2014; Chang *et al.* 2013). The A+T content of protein-coding genes (60.6%) and control region (66.5%) differ by a slight 5.9%. Interestingly, the overall base composition of the entire mitochondrial genome and overall protein-coding genes did not deviate much from each other: 34.0% for A, 25.2% for C, 14.8% for G, 26% for T in terms of overall genome; 33.7% for A, 25.9% for C, 13.4% for G, 26.9% for T in total of 13 protein-coding genes.

### Protein-Coding Gene Features

The gene group that made up almost 68.3% of the entire *T. pauciperforatum* mitochondrial genome is none other than the protein-coding gene group with a total of 11,412 bp coverage over 13 genes. With the translation capacity of up to 3801 amino acids, the protein-coding gene group incorporates genes with size ranging between 165 bp (ATP8) and 1830 bp (ND5). All three overlaps found in this group are located on the H-strand.

The start codon usage of all 12 protein-coding genes are generally ATG, except for the GTG which is found exclusively in COI gene. These phenomena can be seen commonly occurring in *Brama japonica*, *R. steineri*, *R. trilineata*, *R. argyrotaenia*, *R. borapetensis*, *R. aprotaenia* and *R. lateristriata* (Chen *et al.* 2016; Chang *et al.* 2013; Kusuma *et al.* 2017; Ho *et al.* 2014; Zhang *et al.* 2014; Kusuma & Kumazawa 2015). Looking at the termination codon usage, TAA is used by ND1, COI, ATP8, ND4L, ND5 and Cytb; TAG is utilised by ND6; whereas the others (ND2, COII, ATP6, COIII, ND3 and ND4) terminate with incomplete codons. This stop codon pattern is similar as seen in *R. steineri* (Chang *et al.* 2013). However, the termination codon usage is slightly varied across *B. japonica*, *R. trilineata*, *R. argyrotaenia*, *R. borapetensis*, *R. aprotaenia* and *R. lateristriata* (Chen *et al.* 2016; Kusuma *et al.* 2017; Ho *et al.* 2014; Zhang *et al.* 2014; Kusuma & Kumazawa 2015) and this dissimilarity is deemed typical among the vertebrate mitogenomes (Ojala *et al.* 1981). The base composition of all protein-coding genes is depicted in Table 3.

### Transfer and Ribosomal RNA Gene Features

Out of the 22 tRNA genes identifies in this study, 63.6% (14) of them are encoded by H-strand while L-strand is responsible for encoding the other 8 tRNA genes. The anti-codons of all tRNA genes are highly conserved across other fish metagenome such as *R. borapetensis* and *B. japonica* (Zhang *et al.* 2014; Chen *et al.* 2016). The 22 tRNA genes made up nucleotide length of 1552 bp with A+T content of 57.1%, the tRNA^Ala^ topped the group with A+T content of 69.2% whereas the tRNA^Thr^ bottomed the list with A+T content of 48.6%.

Occupying a sum of 15.7% (2624 bp) of the entire mitochondrial genome of *T. pauciperforatum*, both rRNA genes (12S rRNA and 16S rRNA) are 71 bp apart on the H-strand with tRNA^Val^ gene sandwiched in between them. The A+T content of 16S rRNA gene (58.1%) is slightly greater than that of 12S rRNA gene (54.2%), both contributing to the overall total rRNA A+T content of 56.6% and base composition as displayed in Table 3: 35.9% for A, 23.7% for C, 19.6% for G and 20.7% for T.

### Non-Coding Region

**Figure 2:**
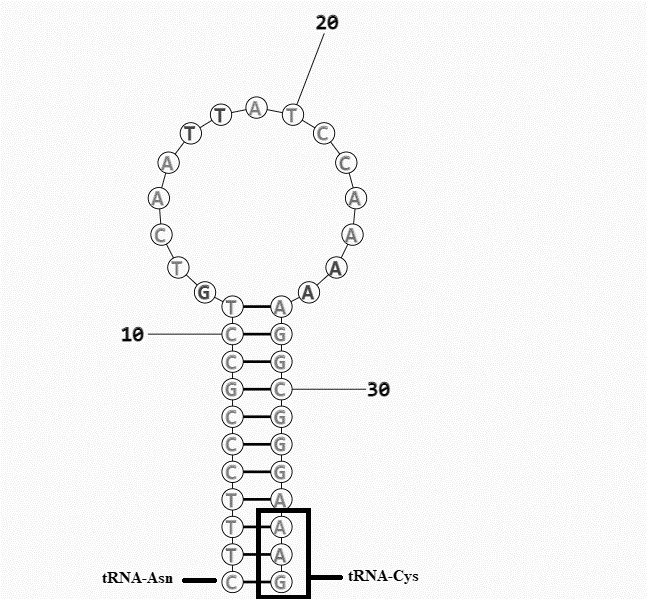
The predicted secondary structure of light strand origin which is situated between tRNA^Asn^ and tRNA^Cys^ genes of *R. T. pauciperforatum*. The part of the tRNA^Cys^ gene sequence is in the box.

Excluding the light strand origin and control region, the other non-coding regions are relatively miniature from 1 to 11 bp. The light strand origin (O_L_) and the control region are the two large non-coding regions to be highlighted among the 16 non-coding regions identified. The light strand origin was located between tRNA^Asn^ and tRNA^Cys^ in the *T. pauciperforatum* mitochondrial genome. This 37 bp region has the stem-loop secondary structure forming capability with the allocation of 11 complementary nucleotide pairs contributing to the stem whilst the loop conformation takes up to 15 nucleotides arranged in closed circle (Fig. 2).

The largest non-coding region of the *T. pauciperforatum* mitochondrial genome, the control region, has A+T content of 66.5%, depicting higher A+T content than that of the overall mitogenome (60.0%), which was similarly detected in mitogenome of *B. japonica* (Chen *et al*. 2016). On the side note, the base composition of this control region is as below: 34.0% for A, 20.9% for C, 12.6% for G and 32.5% for T respectively as shown in Table 3. Besides, the terminal associated sequence (TAS), central conserved sequence block (CSB-F, CSB-D and CSB-E) as well as variable sequence block (CSB-1, CSB-2 and CSB-3) were all traced within the control region of this species.

### Phylogenetic Relationship Analysis

**Figure 3:**
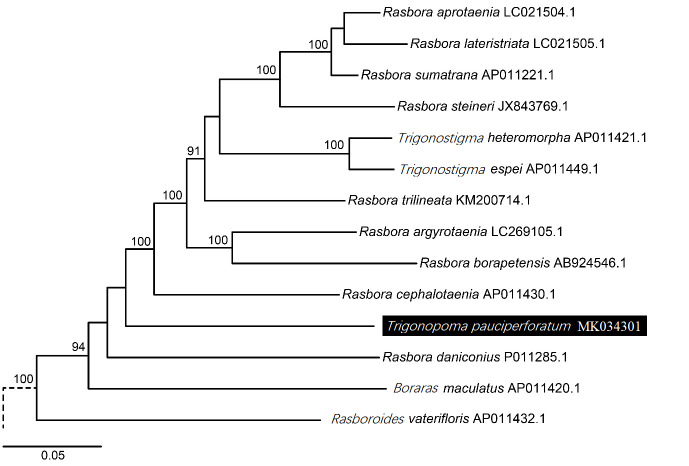
Phylogenetic tree of *T. pauciperforatum* with other *Rasbora* genus members and outgroups, based on 12 protein-coding genes (except ND6 gene) via the GTR+G (General Time Reversible model with Gamma distributed rates among sites) Maximum Likelihood (ML) analysis with bootstrap of 1000 replicates. The tree was rooted (represented by dashed line) by the outgroups *Acheilognathus typus* and *Danio rerio.*

A maximum likelihood tree was constructed to unravel the phylogenetic relationship of *T. pauciperforatum* and its closely related species with the whole mitogenome now available (Fig. 3). The *R. aprotaenia*, *R. lateristriata*, *R. sumatrana* and *R. steineri* form a distinctive cluster with bootstrap value of 100%. Besides, the *T. heteromorpha* and *T. espei* pair as well as the *R. argyrotaenia* and *R. borapetensis* pair also scored 100% bootstrap possibilities which also in agreement to the findings by Kusuma and Kumazawa (2015) as well as Kusuma *et al.* (2017). *T. pauciperforatum* diverged from the basal region of the major clade, where its evolutionary relationships with *B. maculatus*, *R. cephalotaenia* and *R. daniconius* are poorly resolved as suggested by the low bootstrap values there. The phylogeny is rooted (indicated by the dashed line) by the outgroups

Comparing to the morphology based phylogenetic tree constructed by Liao *et al.* (2010) on 29 species of *Rasbora* with 41 morphological characters investigated, some distinctive dissimilarities were observed. For instances, *R. lateristriata*, *R. cephalotaenia* and *R. trilineata* were found to share the same clade when characterized morphologically (Liao *et al.* 2010) but that is not the case in this study. The *T. pauciperforatum* reside on the same clade as *T. heteromorpha* and *R. vaterifloris* when scored morphologically but in this study all three of them are located far apart. Some comparisons across the results of these two trees are not possible yet due to the absence of some species in both analysis. *R. borapetensis* was observed to be closely related to *R. rubrodorsalis* and both of them formed clade with *R.* cf. *beauforti* and *R. semilineata* (Liao *et al.* 2010) whereas in this study, *R. borapetensis* is closely related to *R. argyrotaenia* in which *R. argyrotaenia* was not included in the analysis by Liao *et al.* (2010). *T. pauciperforatum* was discovered as the closest neighbour to its only genus counterpart, *T. gracile* beside sharing the clade with other members like *B. brigittae*, *Rasbosoma spilocerca* and *Horadandia atukorali* which four of them were not included in this study because of the lack of the whole mitogenome sequences (Liao *et al.* 2010).

Another comparison of phylogenetic tree was done to that from Kusuma *et al.* (2016) and the input sequences used are COI, Cytb, RAG1 and opsin gene sequences. One of the similarities detected is that *R. lateristriata* was grouped closely with *R. aprotaenia* and *R. sumatrana*. The grouping of *R. borapetensis* and *R. agryrotaenia* inside the same clade is the other similar scenario observed and the only difference is that in the tree constructed by Kusuma *et al.* (2016), *R. dusonensis* was found to be related closer to *R. agryotaenia* than *R. borapetensis*. The tree from Kusuma *et al.* (2016) depicted a strong clade with members like *T. pauciperforatum*, *T. gracile*, *Kottelatia brittani*, *B. merah* and *R. kalbarensis*, with *B. merah* being the closest to *T. pauciperforatum*. However, due to the absence of mitogenome sequences from the abovementioned species that shares the same clade with *T. pauciperforatum*, this analysis cannot be conducted in this study.

## CONCLUSION

The complete mitogenome of *T. pauciperforatum* has been unravelled with the completion of the sequencing and characterisation process. Besides, this study had also revealed the close molecular phylogenetic relationship between this species and 13 other closely related members of the Danioninae subfamily (from *Rasbora* genus and other species previously classified under *Rasbora* genus). This study also serves as an enrichment towards the complete mitochondrial genome count within the *Trigonopoma* genus in terms of evolution and conservation genetics.

## Figures and Tables

**Table 1:** Primers used for the amplification of the *T. pauciperforatum* mitogenome.

Primer name	Primer sequence	T_m_ (°C)	Amplification length (bp)
SF1	GTGCTTCCTCTACACCAC	55.3	8923
SR1	TGATGTTGAGAAGGCTAC		
LF1	CCTATCTTACCGAGAAAG	48.6	9990
LR1	GAGGCCTTCCCATCTAGA		

**Table 2:** Features of the whole *T. pauciperforatum* mitogenome.

Gene	Position (5′-3′)		Codon			Anti-codon	Intergenic nucleotide^b^ (bp)	Strand^c^
	
	Start	End	Start	Stop^a^	Amino acid			
tRNA^Phe^	1	69				GAA	0	H
12S rRNA	70	1020					0	H
tRNA^Val^	1021	1091				TAC	0	H
16S rRNA	1092	2764					0	H
tRNA^Leu (UUA)^	2765	2839				TAA	1	H
ND1	2841	3815	ATG	TAA	325		5	H
tRNA^Ile^	3821	3892				GAT	−2	H
tRNA^Gln^	3961	3891				TTG	1	L
tRNA^Met^	3963	4031				CAT	0	H
ND2	4032	5076	ATG	T--	348		0	H
tRNA^Trp^	5077	5148				TCA	2	H
tRNA^Ala^	5218	5151				TGC	1	L
tRNA^Asn^	5292	5220				GTT	34	L
tRNA^Cys^	5391	5327				GCA	1	L
tRNA^Tyr^	5463	5393				GTA	1	H
COI	5465	7015	GTG	TAA	517		0	H
tRNA^Ser (UCA)^	7086	7016				TGA	1	L
tRNA^Asp^	7088	7157				GTC	6	H
COII	7164	7854	ATG	T--	230		0	H
tRNA^Lys^	7855	7929				TTT	2	H
ATP8	7932	8096	ATG	TAA	55		−7	H
ATP6	8090	8772	ATG	TA-	227		0	H
COIII	8773	9557	ATG	TA-	261		0	H
tRNA^Gly^	9558	9628				TCC	0	H
ND3	9629	9977	ATG	T--	116		0	H
tRNA^Arg^	9978	10047				TCG	0	H
ND4L	10048	10344	ATG	TAA	99		−7	H
ND4	10338	11719	ATG	TA-	460		0	H
tRNA^His^	11720	11788				GTG	0	H
tRNA^Ser (AGC)^	11789	11856				GCT	1	H
tRNA^Leu (CUA)^	11858	11930				TAG	0	H
ND5	11931	13760	ATG	TAA	610		−4	H
ND6	14278	13757	ATG	TAG	174		0	L
tRNA^Glu^	14347	14279				TTC	6	L
Cytb	14354	15490	ATG	TAA	379		4	H
tRNA^Thr^	15495	15564				TGT	11	H
tRNA^Pro^	15645	15576				TGG	0	L
D-loop	15646	16707						-

Notes:

a TA- and T-- indicate incomplete stop codons;

b Numbers indicate interspaced nucleotides and negative numbers indicate overlapping nucleotides;

c H and L indicate heavy or light strand respectively.

**Table 3:** The nucleotide base composition of all genes in the *T. pauciperforatum* mitogenome*.*

Region	Base composition (%)				A + T content (%)

	A	C	G	T	
Protein-coding gene					
ND1	34.5	26.7	12.9	25.9	60.4
ND2	38.7	28.1	10.3	22.9	61.6
COI	28.4	24.4	16.8	30.4	58.8
COII	33.7	22.6	15.8	27.9	61.6
ATP8	35.8	24.2	8.5	31.5	67.3
ATP6	33.7	25.5	11.1	29.7	63.4
COIII	30.4	25.7	16.2	27.6	58.0
ND3	30.1	27.2	14.3	28.4	58.5
ND4L	29.0	27.6	13.8	29.6	58.6
ND4	33.6	26.3	12.8	27.3	60.9
ND5	35.8	25.2	12.4	26.5	62.3
ND6	44.6	29.9	10.7	14.8	59.4
Cytb	31.7	26.0	14.1	28.2	59.9
Overall of protein-coding gene	33.7	25.9	13.4	26.9	60.6
tRNA gene					
tRNA^Phe^	37.7	20.3	20.3	21.7	59.4
tRNA^Val^	28.2	25.4	23.9	22.5	50.7
tRNA^Leu (UUA)^	28.0	24.0	22.7	25.3	53.3
tRNA^Ile^	25.0	22.2	26.4	26.4	51.4
tRNA^Gln^	35.2	25.4	14.1	25.4	60.6
tRNA^Met^	31.9	30.4	15.9	21.7	53.6
tRNA^Trp^	36.1	22.2	22.2	19.4	55.5
tRNA^Ala^	36.8	22.1	8.8	32.4	69.2
tRNA^Asn^	32.9	27.4	19.2	20.5	53.4
tRNA^Cys^	29.2	27.7	23.1	20.0	49.2
tRNA^Tyr^	31.0	31.0	19.7	18.3	49.3
tRNA^Ser (UCA)^	26.8	28.2	19.7	25.4	52.2
tRNA^Asp^	37.1	20.0	14.3	28.6	65.7
tRNA^Lys^	34.7	25.3	18.7	21.3	56.0
tRNA^Gly^	36.6	22.5	12.7	28.2	64.8
tRNA^Arg^	27.1	25.7	21.4	25.7	52.8
tRNA^His^	34.8	23.2	13.0	29.0	63.8
tRNA^Ser (AGC)^	35.3	19.1	19.1	26.5	61.8
tRNA^Leu (CUA)^	36.5	17.66	17.6	28.4	64.9
tRNA^Glu^	34.8	23.2	17.4	24.6	59.4
tRNA^Thr^	28.6	28.6	22.9	20.0	48.6
tRNA^Pro^	37.1	28.6	11.4	22.9	60.0
Overall of tRNA gene	32.8	24.5	18.4	24.3	57.1
rRNA gene					
12S rRNA	33.9	25.0	20.8	20.3	54.2
16S rRNA	37.1	23.0	18.9	21.0	58.1
Overall of rRNA gene	35.9	23.7	19.6	20.7	56.6
Control region	34.0	20.9	12.6	32.5	66.5
Overall of the genome	34.0	25.2	14.8	26.0	60.0
